# The Pattern of Clinical Activities in Alliance CAMHS Over a 3-Month Period: 2020 vs 2021

**DOI:** 10.1192/bjo.2022.263

**Published:** 2022-06-20

**Authors:** Olajide Adegbite, Tania Saour, Sahar Bhatti

**Affiliations:** West London NHS Foundation Trust, London, United Kingdom

## Abstract

**Aims:**

This Quality improvement project will look into the data collected over the same period in 2020 and 2021 to highlight patterns and changes as a result of the COVID-19 pandemic, and how to improve the quality of service provided by the team.

**Methods:**

The record of a total of 349 patients was accessed from the Alliance team spreadsheet and patient electronic records (Rio) between September and November in 2020 and 2021.

The inclusion criteria include:

1. All patients referred to the team
2. All patients managed by the team
3. Patients referred between September and November 2020
4. Patients referred between September and November 2021

Data collected include:

1. Presenting complaint
2. Demographics- gender and race
3. Source of referral
4. Outcome of referral
5. Timeline of first contact after referral

**Results:**

1. The overall number of referrals between September and November 2020 was more than referrals over the same time period in 2021; 188 patients in 2020 and 161 in 2021
2. Of the 188 referred in the 2020 audited period, 55%(102) were from minority ethnic groups compared to 50%(80) in the 2021 audited period. So the number and proportion of minorities requiring mental health support rose due to the impact of COVID pandemic infections, restrictions, and lockdowns.
3. In 2020, the proportion of male patients was 26%(49) compared to 18%(30) in 2021. This is important because the majority of our patients are females which implies that the COVID pandemic had a significant effect on the entire population leading to more male patient referrals.
4. The overall number of patients that presented with self-harm was greater in 2020 than in the 2021 period of audit.
5. The overall number of patients that presented with anxiety was also greater in 2020 than in the 2021 period of audit.
6. Of the 188 patients referred between September to November 2020, 58% (109) of them were seen within 24 hours of referral compared to 61% (99) in 2021. In the 2021 period, the restrictions have stopped and it has become far easier to carry out assessments at home and school while using the necessary protective gear.
7. It was noticed that there was a lot of telephone support in 2020 but none in 2021. The majority of these patients were those who were already known to the service and were being supported but deteriorated mentally during the peak of the pandemic.
8. There was a lot of referral from the single point of access (SPA) in between September and November 2020 while there was none over the same time period in 2021. This could have resulted from another impact of the pandemic when a lot of service providers were off sick and their patients could not reach them directly so they opted to go through SPA. Some new referrals also came this way.
9. It is also noteworthy that 59% (112) of patients seen in the 2020 audited period were already known to the service while 54%(88) seen in 2021 were known. This implies that a lot of our patients deteriorated due to the pandemic
10. We also had more new referrals in 2021 than in 2020 for the same audited period.
11. Six percent of the 188 patients seen 2020 audited period had telephone support while none did in 2021. Since all restrictions were lifted in July 2021, the service has opted for a more conventional approach of patient assessment which is face to face especially when expedient.
12. Fifty-two percent (85) of 161 patients seen in the 2021 audited period were signposted to another service while 44% (72) of 188 seen in 2020 were signposted.

**Conclusion:**

This audit has proven that not only did the pandemic affect the overall volume of patients seen, but it also increased the proportion of male patients seen and the relative proportion of minority ethnic groups that used the service.

The pandemic and government policies also influenced how patients were assessed seeing how 2020 had a lot of telephone support.

It's impressive to know that the team managed to cope in these challenging periods without compromising the quality and standard of care as well as leaving behind an up to date medical records making this audit possible and easy

Important Recommendations includes:

1. Completing annual audits on the pattern of clinical activities
2. Continued review of quality and consistency of data collection
3. To consider an alternative method for data collection to minimize the risk of human error.
4. Regular training sessions for mental health crisis team in keeping with changes to mental health presentations during the COVID Pandemic.
5. To review data collected and expand on the information collected to include gender and ethnicity

